# Group‐specific environmental sequencing reveals high levels of ecological heterogeneity across the microsporidian radiation

**DOI:** 10.1111/1758-2229.12642

**Published:** 2018-05-17

**Authors:** Bryony A. P. Williams, Kristina M. Hamilton, Meredith D. Jones, David Bass

**Affiliations:** ^1^ Biosciences, University of Exeter, Geoffrey Pope Building Exeter, EX4 4QD UK; ^2^ The Natural History Museum, Cromwell Road London Kensington, SW7 5BD UK; ^3^ Centre for Environment, Fisheries and Aquaculture Science (Cefas), Barrack Road Weymouth Dorset, DT4 8UB UK

## Abstract

The description of diversity is a key imperative in current biological studies and has been revolutionised by the molecular era that allows easy access to microbial diversity not visible to the naked eye. Broadly targeted SSU rRNA gene amplicon studies of diverse environmental habitats continue to reveal new microbial eukaryotic diversity. However, some eukaryotic lineages, particularly parasites, have divergent SSU sequences, and are therefore undersampled or excluded by the methodologies used for SSU studies. One such group is the Microsporidia, which have particularly divergent SSU sequences and are rarely detected in even large‐scale amplicon studies. This is a serious omission as microsporidia are diverse and important parasites of humans and other animals of socio‐economic importance. Whilst estimates of other microbial diversity are expanding, our knowledge of true microsporidian diversity has remained largely static. In this work, we have combined high throughput sequencing, broad environmental sampling and microsporidian‐specific primers to broaden our understanding of the evolutionary diversity of the Microsporidia. Mapping our new sequences onto a tree of known microsporidian diversity we uncover new diversity across all areas of the microsporidian tree and uncover clades dominated by novel sequences, with no close described relatives.

## Introduction

Microsporidia are a diverse phylum of eukaryotic parasites related to fungi, causing important socio‐economic infections (Keeling and Fast, 2002). In the range of 1300–1500 species have been described, with most infections found in invertebrates (Vavra and Lukes, 2013). However, microsporidia also cause infections of immunocompromised patients with the potential to cause loss of life (Weiss, 2014). Seventeen species are now known to cause human infection and many animal lineages appear to provide zoonotic sources for microsporidiosis (Fayer and Santin‐Duran, 2014). Microsporidia are also important agricultural and aquacultural parasites, being responsible for very significant yield‐limiting infections in farmed shrimp (Rajendran *et al*., 2016; Thitamadee *et al*., 2016), and have been suggested to work synergistically with pesticides to increase honeybee vulnerability to colony collapse disorder, demonstrating the potential of microsporidia to exploit changing host vulnerabilities to create new agricultural problems (Cox‐Foster *et al*., 2007; Stentiford *et al*., 2016). They have a worldwide distribution in all major habitats and the phylum encompasses a large amount of undiscovered diversity (Zhu *et al*., 1993; Elizabeth McClymont *et al*., 2005; Ardila‐Garcia *et al*., 2013). For example, an environmental survey of ten species of freshwater snails revealed four new species of microsporidia (Elizabeth McClymont *et al*., 2005), a survey of microsporidian diversity in soil, sand and compost revealed 22 novel microsporidian sequences (Ardila‐Garcia *et al*., 2013) and a survey of wild nematodes uncovered 11 new species of microsporidia (Zhu *et al*., 1993). Furthermore, multiple new lineages are being described within the Opisthosporidia, a newly created superphylum that encompasses the Microsporidia, the Cryptomycota (Syn Rozellomycota) and the Aphelida (Lara *et al*., 2010; Corsaro *et al*., 2014; Karpov *et al*., 2014). Some of these newly described lineages have clear morphological similarities to the Microsporidia and their exact relationship to them remains to be fully established. This suggests that although microsporidia are recognised as a socio‐economically important parasite group, little is known about their true evolutionary diversity and their relative distribution across distinct habitats.

Known microsporidian SSU diversity has been phylogenetically analysed to generate a broad classification of microsporidia based on their branching in five major clades, referred to by number (Vossbrinck and Debrunner‐Vossbrinck, 2005; Vossbrinck *et al*., 2014). These clades have been associated into higher level groups based on the habitat in which their constituent members occur: Marinosporida (Clade 5), Terresporidia (Clades 4 and 2) and Aquasporidia (Clades 1 and 3), based on the assumption of a clear association between phylogeny and host habitat (Vossbrinck *et al*., 2014). However, within each of these major groups there are several exceptions to this association. The SSU data for these phylogenies are largely retrieved by sampling targeted hosts, which themselves have habitat preferences. As a result, this approach may lead to an incomplete picture of microsporidian diversity, limited by sampling only one aspect of the parasites' lifecycles, and by the relatively labour‐intensive method of investigating host individuals for possible infections.

SSU amplicon studies using broadly targeted eukaryotic primers do not amplify microsporidia, because they are extremely genetically divergent. Additionally, many of these studies use a size filtration method to remove small animals, which are likely to be a major reservoir of microsporidian diversity and many DNA extraction methods may not be harsh enough to disrupt the environmentally resistant spores (Bass *et al*., 2007; Lefèvre *et al*., 2008; Massana *et al*., 2015). Therefore, in this study we collected invertebrates and microbes across multiple environments in the United Kingdom across four seasons. We size‐fractionated these samples, extracted DNA and applied microsporidian‐specific primers with the aim of retrieving a broad diversity of novel microsporidian OTUs. We used the universal microsporidian V1F and 530R primer set to amplify an approximately 400 bp stretch of the SSU gene. We also use the same primer sets to screen DNA samples collected during the course of the pan‐European marine microbial eukaryotic sampling effort [http://www.biomarks.eu/; (Logares *et al*., 2014)]. This revealed novel diversity across all five microsporidian clades. Whilst some of this diversity is represented by unique sequences closely related to described microsporidia, other detected lineages clustered with themselves or other environmental sequences that have no close described relatives. This demonstrates that at least some parts of the microsporidian phylogeny are highly undersampled. In addition, we find that our environmental samples do not always fall into the expected ecological classifications (Aquasporidia for aquatic samples, Terresporidia for terrestrial samples and Marinosporidia for marine samples). This non‐correspondence between phylogeny and sampling environment is suggestive of more frequent transitions between different hosts and biomes than previously recognised.

## Results and discussion

**Figure 1:**
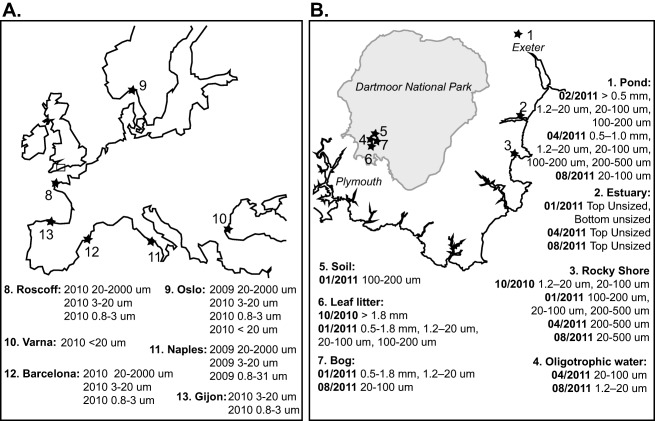
Sample collection sites. **A.** BioMarKs Sample sites across Europe with collection dates and sample sizes. Full details of sample collection methods are available from the BioMarKs website (http://biomarks.eu/?q=fieldwork). **B.** Samples sites within the UK and sample collection dates for which we amplified PCR bands of microsporidan size. The area shown in B is marked as a rectangle on A. Sampling conditions, coordinates and dates are given in Supporting Information Table S1. Several replicate DNA extractions were generated for each sample, but these were eventually pooled before final PCR amplification. Samples were amplified with the microsporidian primer set V1F CACCAGGTTGATTCTGCCTGAC (Zhu *et al*., 1993) and 530R CCGCGGCKGCTGGCAC (Baker *et al*., 1995). using Phusion® High‐Fidelity DNA Polymerase (New England Biolabs) at an annealing temperature of 67°C for 35 cycles. Selected successful BioMarKs amplicons were cloned using the TOPO TA cloning system and sequenced using Sanger sequencing. Several clones were sequenced for each amplicon and vector and primers were trimmed from the clones using Sequencher (Genecodes) before analysis. Samples were also amplified at an annealing temperature of 55°C using primers containing 454 barcodes and adapters and submitted for 454 sequencing.

Multiple DNA extractions were carried out from our UK sample sites, shown in Fig. 1, over four seasons (see Supporting Information Table S1) and different DNA extractions for the same site and collection date were pooled before PCR. However, only a subset of the screened samples (32/109) yielded PCR products with bands that could potentially correspond to microsporidian sequences. These samples are listed as a part of Fig. 1. For sequencing, organismal size fractions from each season were pooled to make a total of 17 pools (See Supporting Information Table S2); after sequencing and removal of non‐target species sequences, 10 of these pools yielded true positive (microsporidian) sequences. Each of the 14 screened BioMarKs DNA samples yielded a PCR band of appropriate size (sampling sites and dates for these are shown in Fig. 1). After sequencing and removal of non‐target species sequences 7 of the 14 samples yielded microsporidian sequences.

**Figure 2:**
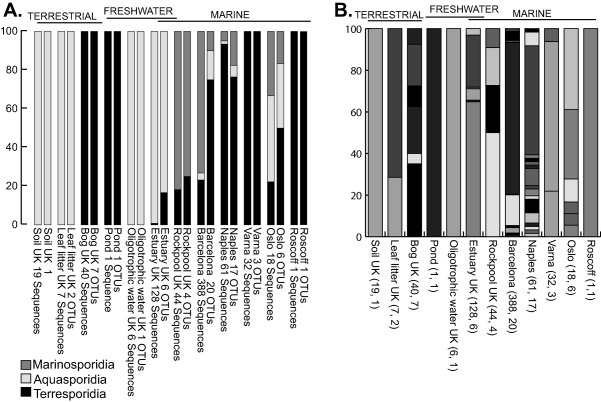
Diversity of sampled microsporidia sequences and OTUs from the sample sites. Sequences were quality filtered and sorted according to barcode using Qiime (Caporaso *et al*., 2010). This resulted in 4746 sequences for BioMarKs samples and 6856 for UK samples. After filtering of obvious non‐target sequences (Those that had very high sequence similarity to non‐microsporidian taxa and tandem repeats), sequences were submitted to Blast2go to identify 438 microsporidian derived sequences for BioMarKs sample and 243 for UK samples (Conesa *et al*., 2005). These were combined into a single file with 64 sequences derived from Sanger sequencing. OTU picking for the whole data set of 745 sequences was carried out using UCLUST in MacQiime which resulted in 58 OTUS. These were screened for chimeric sequences using DECIPHER (Wright *et al*., 2012). **A.** Sequences and unique OTUs classified according to whether they fall within the Aquasporidia (Clades I and III), Terresporidia (Clades II and IV) or Marinosporidia (Clade V). **B.** Diversity detected at each sample site. Each detected OTU is illustrated by a different shade. Number of sequences and number of OTUs per site are given in brackets.

A total of 438 microsporidian sequences were obtained from the BioMarKs samples and 243 from our UK samples, which represented 58 different OTUs. About 17 of these were found in the UK samples and 42 were found in BioMarKs samples. One was found in both sample sets. One sample, Barcelona 20–2000 μm, yielded 388 sequences composed of 20 different OTUS, whilst other sites yielded a single OTU from a single sequence (Fig. 2B). No diversity indices were calculated due to a different sampling effort across the sites.

**Figure 3:**
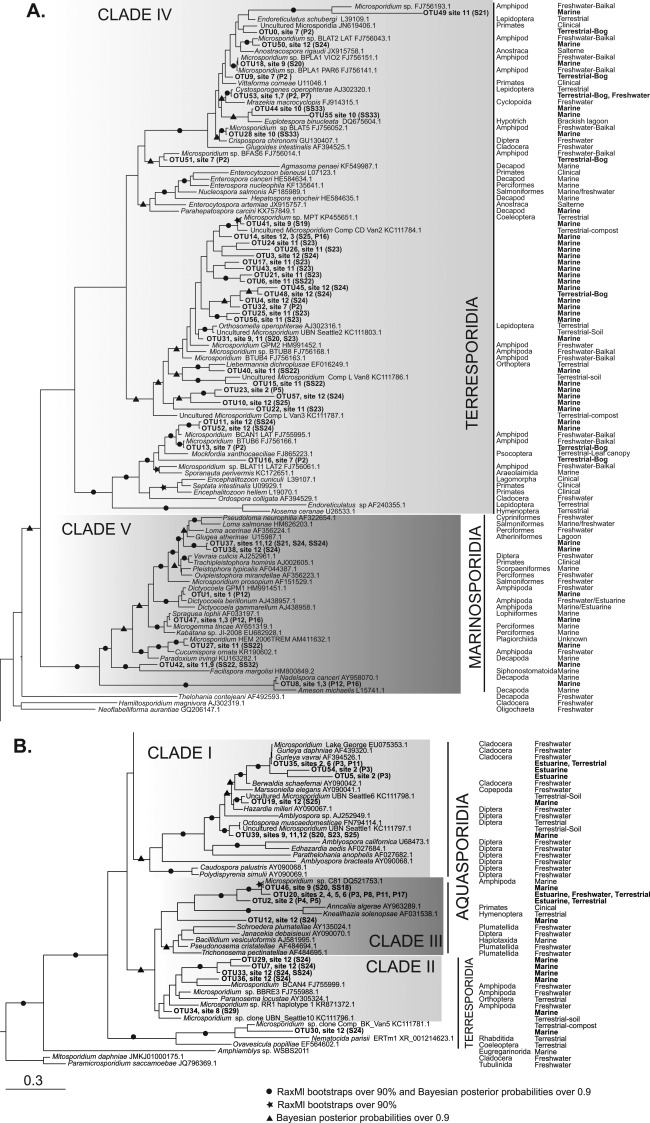
**A, B**. Broad Bayesian phylogeny of new OTUs. Retrieved OTUs were used to query the NCBI database using BlastN and the top hit for each sequence was retrieved. These were added to the OTUS and a reference set of described microsporidia, aligned using MAFFT version 7 set to E‐INS‐i (Katoh and Standley, 2013) and masked using Gblocks (Talavera and Castresana, 2007). Bootstrapped maximum likelihood (ML) analyses were performed using the RAxML BlackBox v. 8.2.8 server (Stamatakis, 2014; Stamatakis *et al*., 2008) on the CIPRES Science Gateway v. 3.3 (Ma *et al*., 2010) Bayesian consensus trees were constructed using MrBayes v 3.2.5 (Ronquist *et al*., 2012) using two MC^3^ runs with randomly generated starting trees for 5 million generations, each with one cold and three heated chains, and using an evolutionary model that included a GTR substitution matrix, a four‐category auto‐correlated gamma correction and the covarion model. All parameters were estimated from the data. Trees were sampled every 1000 generations. The first 1.25 million generations were discarded as burn‐in and a consensus tree was constructed from the remaining sample. To the right hand side of each OTU is indicated the site(s) (see Fig. 1) at which the sequence was found and in brackets either the sample number for BioMarKs samples of Pool number for UK samples (see Supporting Information Table S2 for details of pools and samples). In columns to the right hand side of each branch are the host order (where known) and the host habitat (where known), or the sampling environment for environmental sequences (in bold).

Novel sequences resulting from our current study were added to a broad selection of microsporidian sequences including representatives from all five microsporidian clades (therefore encompassing Terresporidia, Aquasporidia and Marinosporidia). Our phylogenetic analysis largely retrieved these previously recognised ecological clades (Fig. 3A and B) (with two clades corresponding to Terresporidia) and all our novel sequences fell into these clades with no major new clades discovered. Novel sequences were distributed across the tree of microsporidian diversity with little pattern according to environment type, and with new taxa not necessarily falling into the expected clades given their sampling sites (Fig. 3A and B). This non‐correspondence of our results with the Terresporidia, Aquasporidia and Marinosporidia classification system is shown clearly in Fig. 2A.

**Figure 4:**
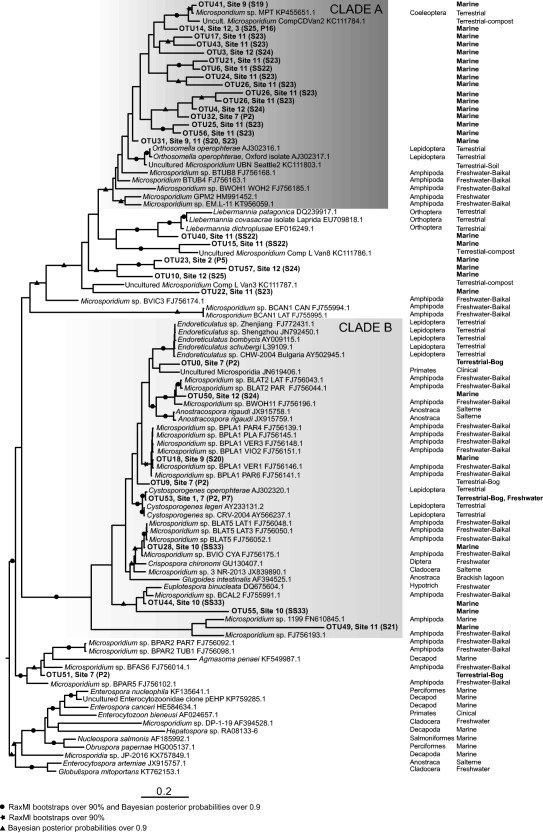
Expanded Bayesian phylogeny of clade IV of the Terresporidia including our retrieved OTUs. The new OTUS from clade IV were used to query NCBI by BlastN again and a new alignment created using these taxa and the top 2 blast hits for each taxon. Sequences that were near identical strains of the same described species were reduced to retain a single sequence. Phylogenies were generated using the methods listed in Fig. 3. To the right hand side of each OTU is indicated the site(s) (see Fig. 1) at which the sequence was found and in brackets either the sample number for BioMarKs samples of Pool number for UK samples (see Supporting Information Table S2 for details of pools and samples). In columns to the right hand side of each species are the host order (where known) and the host habitat (where known), or the sampling environment for environmental sequences (in bold).

Thirty six out of our 58 novel sequences fell into clade IV of the ‘Terresporidia’. Within that clade many of the new sequences fell into a sub‐clade that does not include any characterised taxa and these novel lineages are likely to represent new species and new genera. Figure 4 shows an expansion of this region of the tree, including all available distinct related sequences from GenBank. This detailed phylogeny showed that many of the OTUs generated by this study are from marine DNA samples and cluster with microsporidians previously detected in non‐marine invertebrates: the lepidopteran‐infecting genera *Orthosomella* and *Liebermannia*, and an unidentified Coleopteran infection (Fig. 4, clade A). This phylogeny also shows marine and bog‐derived OTUS from this study within a clade that is dominated by amphipod‐derived samples resulting from a single study of Lake Baikal (Fig. 4, clade B). One explanation is that this particular area (Clades A and B of Fig. 4) of the microsporidian tree is hugely undersampled and wherever we look in the environment, we will find new members of this clade. Alternatively, microsporidia may have undergone a massive diversification within the amphipods and our marine and bog microsporidian samples may all be derived from amphipod hosts and thus be a part of this radiation. However, as this clade also contains lineages from identified insect hosts, perhaps the former explanation is more likely. This clade also contains a microsporidian retrieved from a ciliate host which opens the possibility that our samples were retrieved from protist hosts, such as ciliates, or that protists represent intermediate hosts within this clade. A targeted approach with newly generated clade‐specific primers would be needed to test these ideas and fully explore the diversity for this clade.

In the past, microsporidia have been broadly classified into clades according to their SSU phylogeny and the ecological niche that the majority of species within those clades inhabit (Vossbrinck and Debrunner‐Vossbrinck, 2005; Vossbrinck *et al*., 2014). However whilst it is likely that some microsporidia have a single host, it is also likely that many have a broad host range and that others cycle through unidentified intermediate hosts (Solter, 2014). Nonhost‐targeted environmental screening has the potential to uncover microsporidia in intermediate hosts and environments where they have not previously been documented. This seems to be the case for our results, which have disrupted the pattern of taxa grouping according to environment that they or their hosts are found in.

The majority of our OTUs were detected in a single environment type, but a few were found across multiple environments (Supporting Information Table S2). These are OTU20 found in leaf litter, estuarine, soil and freshwater environments, OTU35 found in leaf litter and estuarine environments and OTU53 found in pond and bog environments. OTU20 is found nested with other sequences retrieved from environmental samples. OTU35 is clustered with *Gurleya* species that infect *Daphnia* species. OTU53 branches as sister to *Cytosporogenes orthopterae*, a moth infecting microsporidian. The finding of certain OTUs in several samples from different environment types is also suggestive of multiple hosts for some microsporidian lineages. However in the absence of data to identify hosts, we cannot rule out the possibility that they are found in the same host type inhabiting a broad range of environments, perhaps, for example, ciliates, nematodes or gammarids that could tolerate a range of salinities. An alternative possibility is that some sample sites could have been contaminated by material from other environments, for example marine and estuarine sites could have been contaminated by run‐off from freshwater and terrestrial environments. We find that marine‐derived OTUs make up a large proportion of some of our clades, for example, the Terresporidia. Whilst this reflects the fact that many of the samples that we used were derived from marine environments (18/30), it also makes it less likely that these large numbers of new taxa would be accounted for by contamination from other habitats.

In both our comprehensive phylogeny and sub‐phylogeny (Figs. 3A, B and 4) we interestingly find a novel sequence, OTU0 found in UK bog samples, as sister lineage of an unidentified microsporidian isolated from the urine of a human patient in Thailand (Figs. 3A and 4). This highlights the close evolutionary relationship between clinical and environmental samples in the Microsporidia, and the possible risk of emergence of human infections from environmental sources. One microsporidian clade known from past studies to contain both environmental and clinical species, is the Enterocytozoonidae, which contains the highly prevalent human pathogen *Enterocytozoon bieneusi* and a number of marine organism‐infecting relatives. In the current study, we recovered no novel sequences that fell into this clade (Fig. 3A). Similarly, we found no new diversity within the genus *Encephalitozoon*, home to several mammalian‐infecting pathogens including humans.

The lack of new diversity in these areas of the tree could suggest that these clades contain less undetected environmental diversity, comprise microsporidia which are very host specific, or that we did not sample the habitats harbouring those lineages. It may also be the case that our broadly targeted primers are biased toward certain clades of microsporidia whilst excluding others. This may be particularly the case for those lineages that are at the base of the known microsporidian diversity and represent potential transitional lineages between the newly characterised taxa such as *Mitosporidium*, *Paramicrosporidium*, *Nucleophaga*, *Amphiamblys* and the main microsporidian diversification. Our single primer approach provides a hint of the environmental diversity waiting to be uncovered in the microsporidia, but a more comprehensive survey of microsporidian diversity will need a larger scale screen with multiple primer sets each targeted at a specific microsporidian clade.

The authors declare that they have no conflicts of interest related to this article.

## Availability of data

New sequences have been deposited at NCBI under accession numbers MG241384–MG241441.

## Supporting information

Additional Supporting Information may be found in the online version of this article at the publisher's web‐site:

**Table S1.** Samples collection details with extraction protocols and sites.

**Table S2.** UK (Sheet 1) and BioMarKs (Sheet 2) DNA pools for 454 and Sanger sequencing (latter for BioMarKs only).
